# Localization of Transient Events Threatening Pipeline Integrity by Fiber-Optic Distributed Acoustic Sensing

**DOI:** 10.3390/s19153322

**Published:** 2019-07-29

**Authors:** Maria-Teresa Hussels, Sebastian Chruscicki, Detlef Arndt, Swen Scheider, Jens Prager, Tobias Homann, Abdel Karim Habib

**Affiliations:** Bundesanstalt für Materialforschung und -prüfung (BAM), Unter den Eichen 87, 12205 Berlin, Germany

**Keywords:** distributed acoustic sensing (DAS), distributed vibrations sensing (DVS), fiber-optic sensing, condition monitoring, pipeline integrity, gas explosion

## Abstract

Pipe integrity is a central concern regarding technical safety, availability, and environmental compliance of industrial plants and pipelines. A condition monitoring system that detects and localizes threats in pipes prior to occurrence of actual structural failure, e.g., leakages, especially needs to target transient events such as impacts on the pipe wall or pressure waves travelling through the medium. In the present work, it is shown that fiber-optic distributed acoustic sensing (DAS) in conjunction with a suitable application geometry of the optical fiber sensor allows to track propagating acoustic waves in the pipeline wall on a fast time-scale. Therefore, short impacts on the pipe may be localized with high fidelity. Moreover, different acoustic modes are identified, and their respective group velocities are in good agreement with theoretical predications. In another set of experiments modeling realistic damage scenarios, we demonstrate that pressure waves following explosions of different gas mixtures in pipes can be observed. Velocities are verified by local piezoelectric pressure transducers. Due to the fully distributed nature of the fiber-optic sensing system, it is possible to record accelerated motions in detail. Therefore, in addition to detection and localization of threatening events for infrastructure monitoring, DAS may provide a powerful tool to study the development of gas explosions in pipes, e.g., investigation of deflagration-to-detonation-transitions (DDT).

## 1. Introduction

In recent years, the integrity of pipelines and piping systems in industrial plants has moved into the focus of public concern as the global piping infrastructure grows and ages. Moreover, to meet increasingly higher standards in technical safety, environmental compliance and failsafe performance the demand for a holistic pipe monitoring system are expected to rise even further in the future. Such a system should ideally be able to detect, classify, and localize a wide range of different threatening pipe conditions prior to the occurrence of leakages over the whole length of the pipeline and in real time. In the long run it should replace time-consuming and costly routine inspections, which are currently taking place at predetermined time-points [1].

Since piping systems in industrial plants typically are highly branched and stretch over long distances the use of conventional point sensors is usually not practicably feasible to realize a monitoring system without gap. Therefore, the principle of distributed fiber optic sensing (DFOS) [1,2] has come into focus for this kind of application as it relies on one single optical fiber applied to the structure under question, which simultaneously acts as a spatially continuous sensor as well as the signal transducer. Hence, the sensor fiber—typically embedded in a measuring cable—may be understood as a chain of thousands of sensors stretching over many kilometers. The effective number of these measurement points is solely limited by the total length to be monitored and the spatial resolution of the measuring technique. As a result, extensive structures can be provided with this type of sensor with comparatively low efforts.

Present DFOS techniques established in pipeline monitoring are widely based on distributed temperature sensing (DTS) and focus on leak detection [3,4,5]. DTS systems allow to identify leakages due to temperature changes induced by escaping gases and fluids (e.g., Joule–Thomson effect). Additionally, distributed strain sensing (DSS) is applied in some cases to detect ground movements and resulting pipe deformations [3,4,5]. However, both techniques belong to the class of so-called static sensing mechanisms meaning that a measurement time of at least several minutes, and up to hours, is required before a change in the condition is detected.

Besides the established methods to measure temperature and strain, distributed acoustic or vibration sensing (DAS/DVS) has lately received considerable attention as a means to detect and localize third party threats to pipelines, i.e., approach of vehicles, digging, and mechanical manipulation on the pipe [3,6,7,8]. The techniques subsumed under the label of DAS/DVS allow the detection and localization of highly dynamic strain changes along the sensor in the kHz regime. Currently, this technology is a matter of continuous development and refinement for special applications both by commercial suppliers as well as in fundamental research. Additional to the above-mentioned application in the oil and gas industry, the great potential of DAS/DVS was also recognized and is currently rapidly evolving in other application fields like perimeter safety [9,10,11], seismic measurements [12,13,14,15,16] as well as train and track monitoring [17,18,19,20].

The principle of distributed acoustic/vibration sensing relies on the detection of Rayleigh backscatter caused by naturally occurring inhomogeneities along an optical fiber by employing the measuring scheme of coherent optical time domain reflectometry (C-OTDR) [21,22]. Basically, as in all implementations of OTDR, a short laser pulse is sent into the fiber under test and the back-reflected light is directed onto a photo-detector, while time-correlation between the initial pulse and the detection allows to deduce the spatial coordinate along the fiber via the speed of light. In the case of coherent OTDR, a highly coherent laser source is employed so that the backscattered light interferes coherently within each portion of the fiber illuminated by the pulse. This leads to the acquisition of a distributed speckle interferogram at the detector, which is highly sensitive to the smallest strain and temperature changes allowing for the acquisition of fiber fingerprints on a shot-to-shot basis. If no further components are employed to disentangle the phase of the recorded signal, the method is called direct detection C-OTDR, the simplest form of DAS/DVS. As the distribution of scatterers within the fiber is inherently random and consequently the transfer function of the distributed interferometer is unknown, such a system is not able to provide any quantitative information on the value of the strain. However, it does allow for the detection and localization of the overall occurrence of dynamic strain changes as well as their frequency. Further data analysis of C-OTDR data using methods of pattern recognition and machine learning has allowed successful recognition and classification of threatening events in a number of studies [23,24,25].

Although, strictly speaking, the term distributed acoustic sensing would imply detection of the full acoustic field including the phase, in many application fields it has become a convention to collect all C-OTDR schemes under the term DAS. For brevity, we will therefore employ this designation in the following.

In the sense of a holistic monitoring system described above, the so far not utilized potential of DAS as a means for continuous condition monitoring of pipes by detecting and localizing acoustic signals that point to certain damage scenarios, has been investigated in the interdisciplinary research project AGIFAMOR (Ageing Infrastructures—Fibre Optic Monitoring of Pipes) at BAM from 2015 to 2018 [26]. The feasibility study has focused on three scenarios of potentially detectable acoustic events:

1. Undamaged pipe: incidents causing (dynamic) circumferential changes of the pipe or initiating propagation of acoustic waves in the pipe wall (e.g., pressure shocks and cavitation, external mechanically induced shocks/vibrations).
2. Damaged pipe: changes inside the pipeline causing altered flow generated noise (corrosion, sedimentation, clogging, in extreme cases also noise caused by leakage).
3. Developing damage of the pipe: crack formation and propagation in the wall with accompanying acoustic emissions.

While the results for scenarios 2 [27] and 3 [26] are described elsewhere, the current study focuses on the early recognition of potentially threatening events according to scenario 1 in the undamaged pipe before occurrence of structural failure.

Since the better part of the events falling into the first category is highly transient, e.g., impacts on the pipe wall or pressure waves travelling through the pipeline, it is necessary to develop a scheme for data analysis that makes do without any temporal averaging. Especially for steel pipes, which are commonly used, localization is additionally aggravated since each event causes acoustic waves propagating inside the pipe wall with the very high speed of sound around 5000 m/s and very little dampening. This means that dynamic strain changes are transmitted to the sensor fiber along many meters along the pipe within milliseconds, which could significantly reduce the spatial resolution of the measuring system. Therefore, tracking of propagating waves on a very fast time-scale is necessary to reveal the signal onset and the location of the initial source of disturbance.

In any implementation of a (distributed) fiber-optic sensing system the application of the optical sensor fiber to the structure to be monitored is crucial. This is especially true for cases, where (dynamic) strain has to be transmitted to the fiber. Nevertheless, in most of the established applications of DFOS for pipeline monitoring, standard optical telecommunication cables are employed which are usually installed in the ground with some spatial gap to the pipeline. This very cost-efficient sensor installation is suitable for detection of temperature changes caused by leakages (DTS) and the above-mentioned detection of third-party intrusion by recognizing vibration patterns transmitted via the ground (DAS). However, to enable a true condition monitoring system that is sensitive to small changes in the piping system itself and amongst others needs to pick up circumferential changes of the pipe, installation of the optical fiber sensor directly on the pipe is a prerequisite.

The optimization of the application method was one of the main aims of the project AGIFAMOR and in a first investigation comparing different ways of linear fiber application along a pipe the importance of a high quality fiber installation was underlined [28]. Additionally, it was found that close contact of the fiber sensor to the pipe along the whole length of the sensor, e.g., gluing of the whole fiber to the pipe, is crucial.

Another way to provide close contact is helical wrapping of the fiber sensor around the pipe. This type of application has already been employed when carrying out DSS measurements on pipes [29,30] and has proven suitable for transmitting strain changes from the pipe to the sensor fiber. Due to the application of more fiber length per pipe length this geometry provides an increased spatial resolution and a higher sensitivity. For the detection of circumferential changes targeted in this study the helical design provides even further sensitivity enhancement as the optical fiber is sensitive to strain changes only along its axial direction. Optimization of fiber-optic cable design with respect to this directionality is currently addressed in in the context of seismic measurements [31,32,33] leading to the deployment of different sensor geometries depending on the propagation mode of the targeted pressure wave.

On the down side, helical application of the optical fiber sensor to the pipe reduces the overall accessible length of pipe and is therefore expected to be of interest for short to medium pipe lengths. Additionally, it is noted that the bandwidth of the system is reduced in comparison to a linear fiber application on the same pipe length as the maximum pulse repetition rate depends inversely on the length of the fiber under test.

However, in the sense of a feasibility study, we focus on optimal conditions with respect to spatial resolution and sensitivity. Therefore, in the work presented here, a bare fiber is used wrapped helically around the pipe under test without any additional glue to exclude influences arising from the adhesive. Two helical configurations are investigated in the following. The first corresponds to a fully distributed sensor system, where the fiber is continuously wrapped around the pipe with a defined pitch, while the second constitutes a quasi-distributed system, where the fiber is applied in spaced segments of densely wound fiber. The latter resembles the deployment of point sensors—as was for example investigated by Cui et al. [34] for acoustic emission transducers—and may be used in otherwise inaccessible regions in a piping system that may be encountered when retrofitting pipes. Additionally, the quasi-continuous setup bears the potential to reach even higher sensitivity for favorable configurations as summation schemes like e.g., the lately proposed adapted method of random quadrature modulation (RQD) [35] may be used. By picking regions of interest and bridging others by linear fibers this scheme may also provide a way to reach longer distances in a piping system while maintaining the advantages of the helical design in crucial areas. Hence, either configuration or a combination of both may be favorable depending on the actual boundaries and requirements in a specific monitoring task.

For a fundamental investigation of the capability of DAS in conjunction with a suitable sensor application geometry to detect and localize threatening pipe conditions in an undamaged pipe, as summarized under scenario 1, the present study was carried out in two sets of experiments.

In the first set of experiments presented in this paper, we compare the fully distributed and quasi-distributed sensor configuration with respect to their capability to localize transient acoustic events occurring on a pipe. As a showcase application mechanical impacts on the outside of the pipe wall are analyzed. Different procedures for data evaluation and automatic impact detection and localization are introduced. Consequently, localization accuracy and precision are analyzed for different sensor configurations, data evaluation procedures and experimental settings of the DAS system.

In the second part an example for monitoring critical conditions occurring inside the pipe, i.e., propagation of pressure waves, is presented. Data evaluation procedures introduced in the first part are employed to detect and localized such events. Additionally, we focus on separation of different acoustic signatures occurring at the same time and capturing of non-linear propagation.

## 2. Materials and Methods

### 2.1. Fiber-Optic Sensing System and Data Evaluation

In all experiments a sensing system was used consisting of a standard single mode optical fiber (SMF-28e+, Corning) without jacketing and a commercial direct detection C-OTDR-interrogator (Helios DAS, Fotech Solutions Ltd., Hampshire, UK). This system is specified for a maximum range of 40 km with optical pulse lengths between 10 ns and 1 µs and has a maximum pulse repetition rate of 100 kHz. The settings chosen in this study are specified for each experiment in the following paragraphs. For optimal signal transduction, the optical fiber was pre-strained and directly applied onto the surface of the pipe under investigation by helical wrapping using an inhouse designed wrapping device. Uniform and defined pre-strain was achieved by employing a loosely hanging fiber spool with an attached weight onto the sensor fiber during the wrapping process.

Optical pulse lengths *T*_p_ used to interrogate the fiber were optimized for each experiment. Longer *T*_p_ entail higher sensitivity but lower spatial resolution $r≅cT_{p}/\left(2n_{\mathrm{eff}}\right)$, where *c* is the speed of light in vacuum and *n*_eff_ is the effective refractive index of the optical fiber mode. Pulse lengths were chosen between 30 ns and 100 ns, corresponding to an expected spatial resolution of approximately 3 m to 10 m along a straight fiber stretch. With respect to localization along the pipe, spatial resolution is additionally increased by a factor given by the pitch of the helically wrapping of the fiber sensor and the outer diameter of the pipe. To capture transient processes on a short time scale with high precision the pulse repetition frequency *f*_p_ was set to 80 kHz—lose to the maximum possible setting of the interrogator—for experiments targeting acoustic waves in the material and to 40 kHz for experiments targeting pressure waves in the medium. According to Nyquist’s theorem, this allows for registering of signals up to 40 kHz or 20 kHz, respectively. It is noted that the maximum monitoring distance *L*_m_ along the optical fiber inversely depends on the repetition frequency given by $L_{m}=c/\left(2n_{\mathrm{eff}}f_{p}\right)$. This restricts the maximal monitoring distance to 1.25 km of optical fiber in this case. However, *f*_p_ may be adjusted to lower values depending on the specific monitoring task and required bandwidth.

Position along the fiber is usually given in bins, where one bin corresponds to the portion of fiber from which the interference pattern is analyzed within the interrogator. With the system used one bin represents either 0.57 m or 0.68 m of fiber depending on the digital sampling settings (180 or 150 mega samples per second) for the two sets of experiments, respectively.

Raw data recorded by the C-OTDR system was further processed using a home-written Python program. Transient events propagating along the pipe appear as edges within the three-dimensional plot of signal intensity versus time and position along the fiber. For optimal detection of these edges, we employ edge filtering by numerical differentiation of raw data along the time axis. Step width for calculating estimated derivatives was optimized for each experiment.

**Figure 1:**
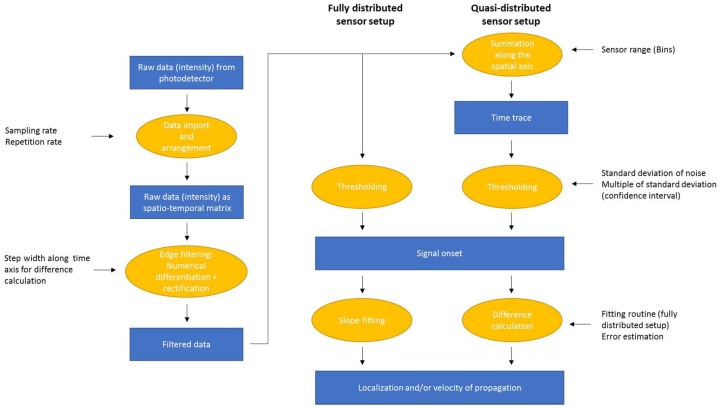
Program flow chart illustrating the procedures used for data evaluation together with influencing variables.

After preparing data in this way, edges were automatically detected by thresholding the signal intensity within a certain bin or summed up intensity within a range of bins with respect to the noise level. The threshold level was set as a multiple of the standard deviation of the noise. From the signal onsets detected in this way the propagation velocity and/or localization of events was deduced by either slope fitting in the case of a fully distributed setup, or by calculation of the runtime difference for the quasi-distributed setup. The whole process of data analysis is summarized in Figure 1.

For sensor ranges that can be approximated as localized sensors, data processing was carried out based on the procedure of random quadrature demodulation (RQD) proposed in [35] for this type of distributed interferometric data. High pass filtering required in RQD was represented here by the data evaluation step of numerical differentiation as this leads to a similar effect. Subsequently, the signal was rectified and summed up over the relevant range of bins.

Numerical simulation of the propagation of acoustic waves in cylindrical waveguides was carried out using the MATLAB toolbox PC disp (Pochhammer–Chree dispersion) [36]. Pipe geometry chosen for simulations was defined by an inner radius of 20.35 mm and an outer radius of 24.15 mm and was embedded in one layer. Assuming steel as the pipe material further parameters used were a bar speed of 5047 m/s, Poisson ratio of 0.3 and a shear modulus of 76.9065 GPa.

### 2.2. Setup for Tracking of Acoustic Waves in the Pipe Wall

**Figure 2:**
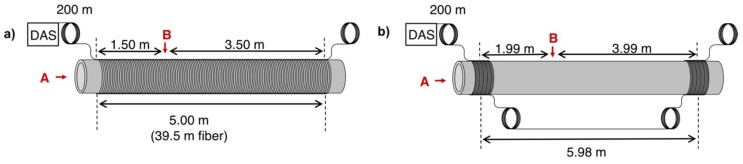
Schematic illustration of the two optical fiber configurations used to track propagating acoustic waves in the pipe wall after excitation by a short mechanical impact. A and B indicate the positions in which mechanical impacts were caused on the pipe. (**a**) Fully distributed sensor setup. (**b**) Quasi-distributed sensor setup with two localized sensor ranges applied to the pipe.

For laboratory-scale tests a stainless-steel pipe of 6.25 m length, inner diameter of 40.7 mm and outer diameter of 48.3 mm was simultaneously equipped with two separate sensor configurations. The pipe was open on both ends. Each configuration could be connected to the C-OTDR system independently. They are schematically depicted in Figure 2.

Configuration 1 represents a fully distributed system, where the fiber is helically wrapped around the pipe on the full length. Pitch of the fiber was 2 cm, so that 7.9 m of fiber correspond to 1 m on the pipe. Altogether 39.5 m of fiber were applied to the pipe in this manner.

Configuration 2 corresponds to a quasi-distributed setup with two localized sensors regions at the beginning and at the end of the pipe. Each of the two localized sensors sections consists of approximately 30 m of fiber densely wrapped around the pipe covering a region of 3.7 cm to 3.8 cm. They are separated by two loose coils of fiber (15 m each), which are decoupled from the pipe, to ensure separation of signals originating from the two sensor regions. The distance between the centers of the two sensor regions amounts to 5.98 m.

In each setup, a coil of 200 m of fiber was introduced between the measuring system and the applied fiber stretch under test.

Short mechanical impacts were produced by manually hitting the pipe with a metallic device either on the front face (A) or on the side (B) as indicated in Figure 2.

### 2.3. Setup for Tracking of Pressure Waves in the Medium

Propagation of pressure waves within the medium inside a pipe was caused by gas explosions in a laboratory-scale gas-filled pipe. The setup for these experiments was placed in an underground bunker dedicated to pipe tests involving explosive gas mixtures. The pipe equipped with sensors and the ignition device was set up in a separate room whereas control of gas filling and ignition as well as data acquisition was carried out remotely. The pipe used for explosion tests was a stainless-steel pipe of 5.54 m length, inner diameter of 30 mm and outer diameter of 38 mm, which was sealed on both ends and contained two sensor penetrations.

**Figure 3:**
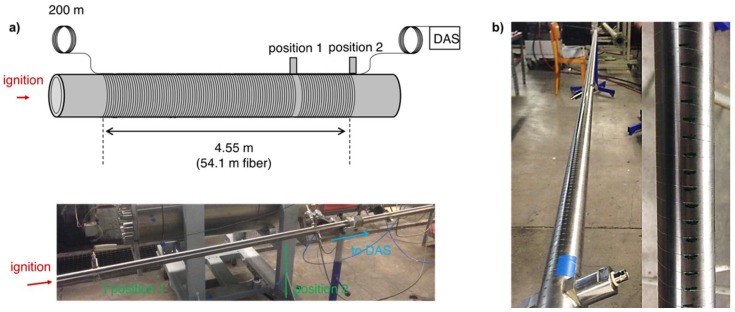
(**a**) Schematic illustration and photo of the optical fiber configuration used in the experiments involving gas explosions in pipes. Positions of the two sensor penetrations for piezoelectric pressure transducers are indicated. (**b**) Detail photos of the optical fiber wrapped helically around the pipe under test.

Figure 3a gives a schematic overview of the instrumented pipe. A continuous configuration of optical fiber was chosen similar to configuration 1 as described above to ensure fully distributed measurements, which are a prerequisite to track accelerated pressure waves. Fiber pitch was 1 cm yielding a total applied fiber length of 54 m on a stretch of 4.55 m of the pipe (detail photo shown in Figure 3b). As a reference, two piezoelectric pressure transducers were placed in the sensor penetrations. Their positions were one meter apart from each other at the end of the region monitored by the optical fiber (Figure 3a).

After evacuation of the pipe different gas mixtures were introduced into the pipe to the desired pressure level (0 to 10 bar overpressure). Consequently, ignition was carried out at one end of the pipe employing a melting wire. Gas mixtures used were 4.2% vol. propane in air and 28.5% vol. hydrogen in air.

Overall guide values of pipe temperature were monitored using a conventional thermocouple placed close to the sensor penetrations on the surface of the pipe. Maximum temperatures throughout the course of the test series amounted to around 50 °C.

## 3. Results

### 3.1. Fully Distributed Detection and Localization of Short Impacts on Pipes

**Figure 4:**
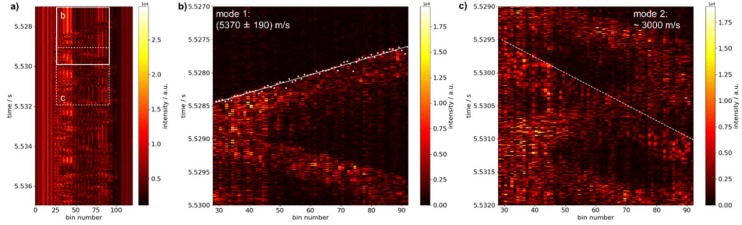
Data evaluation for tracking of propagating acoustic waves using a fully distributed sensor configuration. Excitation took place on the front face (position A). (**a**) Temporal excerpt of the acquired raw data with marked windows of the sections represented in (**b**,**c**); (**b**,**c**) data filtered by differentiation using a temporal shift of 2 and 5, respectively, for improved edge detection. Automatically detected signal onset (white dots) as well as fitted (white solid line) and manually plotted (white dashed line) slopes used for determining the velocities are overlaid.

As a first test of the ability of the system to localize short mechanical impacts on the pipe, the pipe was hit on the front face (Figure 2, position A) while a continuous measurement by C-OTDR was carried out using the fully distributed sensor configuration 1. A pulse length of 100 ns was chosen to achieve high signal intensity at acceptable spatial resolution. Figure 4a shows a temporal excerpt of the acquired raw data. Signal intensity is plotted as a function of time and bin number representing the position along the optical fiber. The signal appears as a zigzag pattern between bins 19 and 101. Considering, the spatial resolution of 10 m along the optical fiber for the chosen pulse length of 100 ns, data outside the region between bins 28 and 92 was discarded for further evaluation. The remaining range of 64 bins or 64×0.57 m=36.5 m corresponds well to the region of fiber which is in close contact to the pipe surface due to the helical wrapping. No signal is visible outside this fiber region, that could be attributed to the mechanical impact. Therefore, the influence of air-borne sound caused by the impact can be neglected and the pattern observed in Figure 4 is understood as the structure-borne acoustic wave traveling in the pipe material, which is reflected from both ends of the pipe segment. It extends the full length of the pipe without any obvious damping and can be observed for several seconds. This underlines the necessity to capture the propagation of the acoustic wave at the onset of the signal on a fast time-scale in order achieve a localization of the event on the pipe.

The slope of the edges appearing in Figure 4 allows to deduce the velocity of signal propagation throughout the pipe material. After filtering of raw data for improved edge detection (differentiation with temporal shift of 2), the signal onset could be automatically detected by setting an appropriate threshold. Points of signal onset are used for linear fitting as shown in Figure 4b. The slope of the fitted line amounts to (5370 ± 190) m/s.

Although propagation with this velocity appears to be dominant over time, a second mode of acoustic propagation with a considerably lower velocity can be discerned. In Figure 4c a temporal excerpt is shown, where manifestation of this mode is hardly overlaid by other signals. However, for this mode, automatic edge detection is not readily obtained. Manually, a velocity of approximately 3000 m/s may be estimated from the slope marked in Figure 4c, which was drawn along the corresponding edge visible in the image. For a pipeline with greater length less reflections of acoustic waves and therefore less crossings of the acoustic signatures are expected. Hence, detection of additional edges like the one observed here may be simplified.

In a second step, a more realistic scenario of impact detection and localization was realized by hitting the pipe in an arbitrary position on the side (Figure 2, position B). Such a scenario may also be encountered in industrial plants with long connected pipe systems. Additionally, this setting allows to assess localization accuracy and precision for different settings of the DAS system, i.e., different optical pulse lengths.

**Figure 5:**
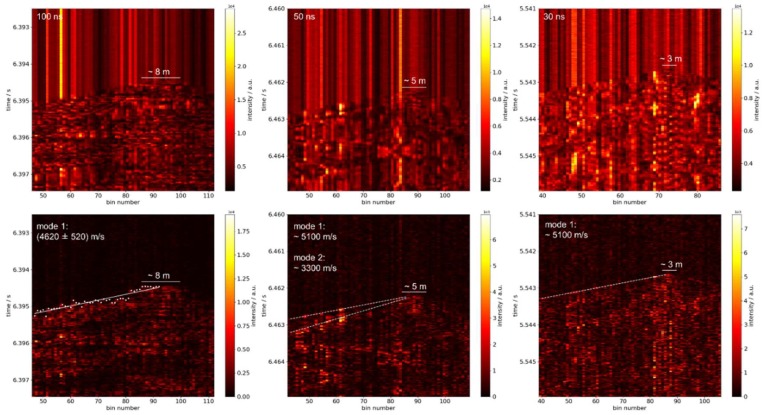
Excerpts of data acquired when hitting the pipe in an arbitrary position on the side (position B) using a fully distributed sensor configuration. The upper row depicts raw data for different optical pulse lengths (100 ns, 50 ns, and 30 ns), while corresponding filtered data is presented in the lower row after differentiation with temporal shifts of 2 (100 ns) and 3 (50 ns, 30 ns). The fiber-optically identified position of impact in the filtered data with associated localization precision is represented by white bars. White dots depict automatically detected signal onsets and white lines show fitted (solid lines) and manually plotted (dashed lines) edges, respectively.

Raw data and filtered data recorded for such events using different pulse lengths are compared in Figure 5. As expected, acoustic waves were observed traveling from a common source to either side of the pipe, where they were reflected. As a result, many crossing signals lead to blurring of the acoustic signatures after the first reflections on the edges.

However, signal onsets, which are the important signal feature for localization of the impacts, are still observable. For 100 ns pulse length, the onset may be determined automatically as in the case of impact in position A described above. Linear fitting results in a propagation velocity of (4620 ± 520) m/s in agreement with the above value within the scope of the measurement uncertainties. The observed greater uncertainty when hitting the side of the pipe may be a result of less effective excitation of the present acoustic modes than for excitation on the front face.

For shorter pulse lengths (50 ns, 30 ns) and impacts in position B the signal-to-noise ratio is not sufficient for automatic edge detection and derivation of velocities. For these cases, acoustic propagation velocities are estimated manually from visual recognition of the edges. The derived values are approximately 5100 m/s for the main mode and 3300 m/s for the second mode using 50 ns pulses and 5100 m/s for the main mode using 30 ns pulses. All of these are in good agreement with the ones derived for position A.

Localization of the source is readily obtained in all cases, as indicated in Figure 5, and localization precision is directly given by the spatial resolution of the measurement system. We find 8 m, 5 m, 3 m, for 100 ns, 50 ns, and 30 ns respectively.

### 3.2. Quasi-Distributed Detection and Localization of Short Impacts on Pipes

**Figure 6:**
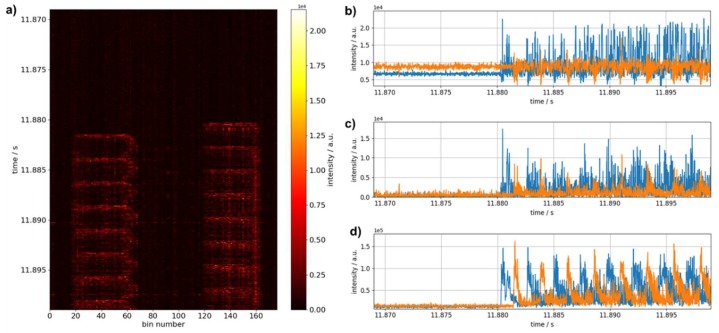
(**a**) Temporal excerpt of filtered data (differentiation with temporal shift of 1) acquired when hitting the pipe on the front face using a quasi-distributed sensor configuration with two localized sensor ranges, (**b**–**d**) time traces recorded by the sensor closer to (blue) and further away (orange) from the point of impact, when observing raw data of one bin, filtered data from the same bin or filtered data summed over the full range of bins relevant to the point sensor, respectively.

Localization of short impacts on a pipe equipped with a quasi-distributed fiber-optic sensor configuration as described in Figure 2b was tested in a similar manner by hitting the pipe mechanically in positions A and B. The data evaluation procedure is exemplarily presented for position A and a pulse length of 50 ns in Figure 6. The two sensor regions are visible in the raw data (Figure 6a) between bin 17 and bin 67 as well as bin 114 and bin 163, corresponding to ranges of 29 m and 28 m. Further processing is carried out in a range between bin 25 and bin 59 and between bin 122 and 155, respectively, to evade influences from the loosely hanging fiber regions.

In each sensor region, the signal is detected simultaneously over all bins and is recurring periodically as the acoustic wave is reflected on both ends of the pipe. Therefore, the two sensor regions indeed act as point sensors with respect to the propagating acoustic waves, and signal processing using summation over the range of each localized sensor (e.g., RQD) is a valid approach. The potential of such an approach is illustrated in Figure 6b–d. While (b), (c) illustrate the temporal evolution of the signal intensity for one bin taken from the center of either region without and with filtering, (d) shows the signal obtained after summation of filtered data over the total range of bins in each region. The latter exhibits a significantly improved signal-to-noise ratio and hence much more expressed signal onset. The time points of signal onset may be readily obtained from these traces by setting an appropriate threshold and the velocity calculated from the time difference and the known distance between the localized sensors is (5320 ± 180) m/s. The measurement uncertainty is given by error propagation of the position uncertainty introduced by the finite length of the two localized sensors and the uncertainty in the determination of the time difference between the two sensor regions.

**Figure 7:**
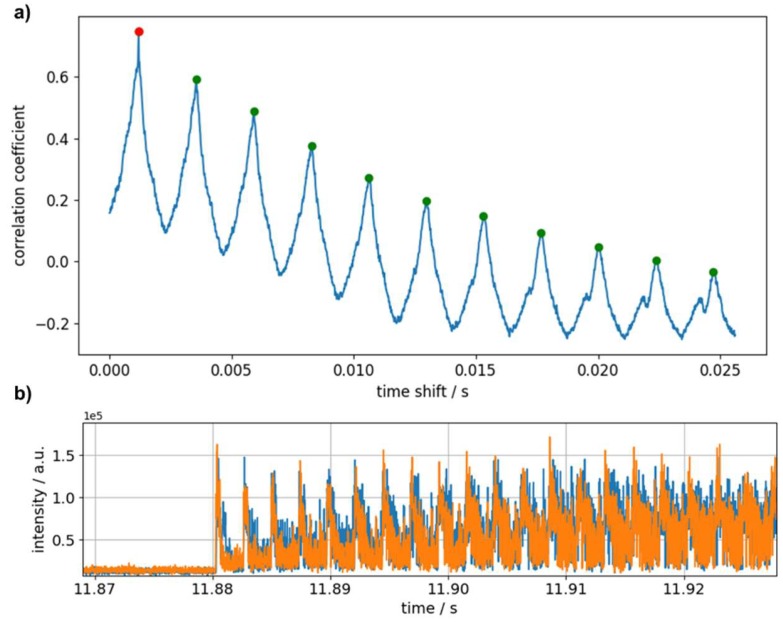
(**a**) Correlation function for the two time traces after filtering and spatial averaging recorded by the sensor closer to (blue) and further away (orange) from the point of impact. Absolute (red) and relative (red and green) maxima of the correlation are marked. (**b**) Corresponding time traces shifted according to maximum correlation.

Additionally, the recurring nature of the signal may be used for data evaluation by correlation of the two time traces presented in Figure 6d. Figure 7a shows the resulting correlation function. At the absolute maximum, the correlation coefficient is 0.78, and a velocity of (5040 ± 170) m/s is deduced for the corresponding time shift. Considering the first eleven maxima of the correlation function a mean value of (5080 ± 170) m/s is derived. Time traces shifted according to this time offset are presented in Figure 7b.

**Figure 8:**
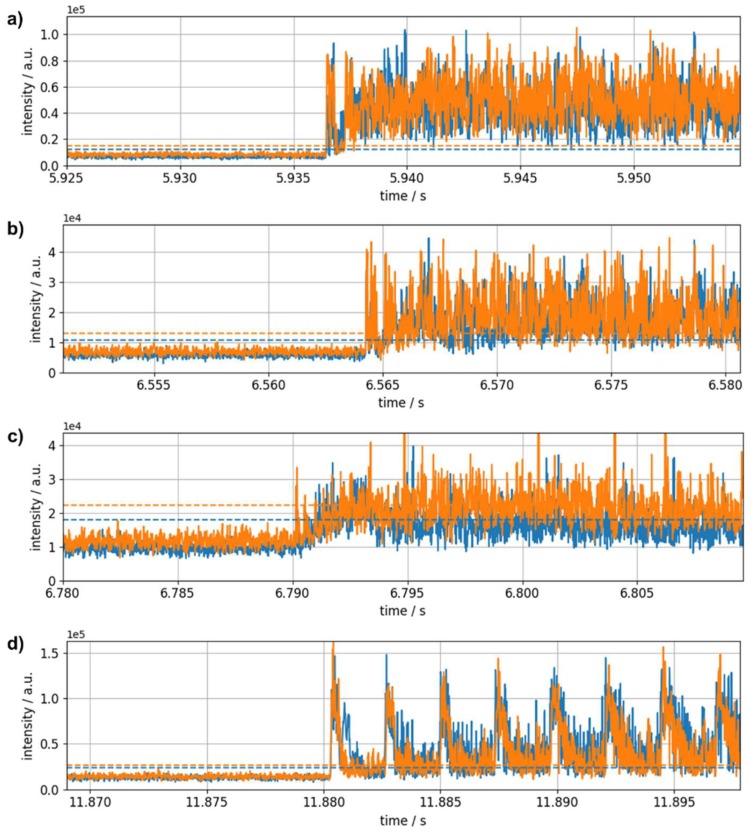
Time traces after filtering and spatial averaging shifted according to the time difference deduced from signal onset for the two localized sensor regions when excitation takes place at the side of the pipe for (**a**) 100 ns, (**b**) 50 ns, and (**c**) 30 ns optical pulse length. (**d**) For comparison shifted time traces for excitation on the front face and 50 ns pulse length are shown. Dashed lines indicate the threshold level used for determining signal onsets.

If excitation takes place on the side of the pipe (Figure 2, position B) the periodicity seen above is not as expressed, which may again be attributed to blurring caused by signal crossings and to a less effective excitation of the acoustic mode resulting in an overall lower signal-to-noise ratio. Therefore, signal onset is used for the determination of velocities. Setting appropriate thresholds, we find (5710 ± 210) m/s, (5510 ± 200) m/s, and (5510 ± 200) m/s using optical pulse lengths of 100 ns, 50 ns, and 30 ns, respectively. Time traces shifted with respect to signal onset are shown together with the ones obtained after excitation in position A (pulse length: 50 ns) in Figure 8.

All velocities determined using the quasi-distributed sensor configuration 2 are compatible with the ones derived using the fully distributed configuration 1.

The propagation velocities deduced for different experimental setups and data evaluation procedures are summarized in Table 1.

### 3.3. Fully Distributed Tracking of Pressure Waves in Pipes

For the second set of experiments, where a scenario of pressure waves in pipes was modelled by inducing gas explosions on one end of the pipe, a fully distributed sensor configuration was chosen to capture the full profile of the propagation. The pulse length was 30 ns for all experiments of this type.

**Figure 9:**
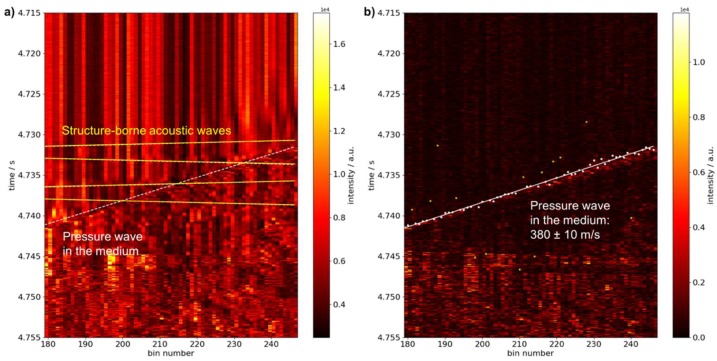
Temporal excerpt of data acquired using a fully distributed fiber-optic sensor configuration after igniting a gas mixture of 4.2% vol. propane in air on one end of a closed pipe at 6 bar initial overpressure. (**a**) Raw data with manually plotted yellow solid and white dashed lines highlighting signal features due to acoustic wave propagation in the pipe material and pressure waves traveling in the medium after ignition, respectively; (**b**) data filtered by differentiation (temporal shift of 4) with white points indicating automatically detected signal onset of the pressure wave (yellow points are discarded as they belong to signal features caused by acoustic waves in steel) and fitted slope (white solid line).

Figure 9 shows the acquired data after igniting a gas mixture of 4.2% vol. propane in air at an initial overpressure of 6 bar from one end of the pipe. Raw data exhibits two different signal progressions with differing propagation velocities as indicated by dashed lines to guide the eye. All signal features are visible in the spatial range between bins 174 to 252 corresponding to a range of 53 m of applied fiber. To discard regions possibly influenced by the loose fiber loops the range of from bin 179 to 247 is used for data evaluation.

The faster component may be attributed to structure-borne acoustic waves travelling inside the pipe material after the explosion, while the slower component should correspond to the pressure wave inside the medium. The signal of the pressure wave is clearly dominant here in accordance with a larger dynamical circumferential strain change than the one caused by the structure-borne waves in steel. However, the dashed lines with a slope of ±5050 m/s matching the expected acoustic velocity reflect well the observed signals in advance of the pressure wave.

To analyze the slower component in greater detail, differentiation with a larger temporal shift is used as a filter. Automatic identification of signal onset reveals some points that are attributed to other signal features (i.e., structure-borne waves) and are discarded for further processing of the data. A linear fit of the remaining points yields a velocity of the pressure wave of (380 ± 10) m/s. The average velocity determined by the two conventional pressure sensors is 406 m/s and is compatible with valued derived by DAS measurements.

**Figure 10:**
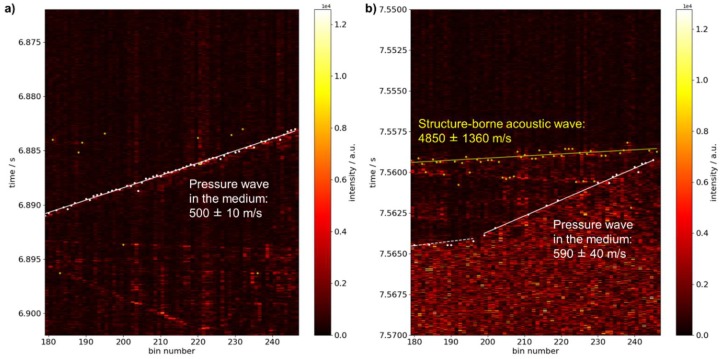
Temporal excerpt of filtered data (differentiation with temporal shift of 4) acquired using a fully distributed fiber-optic sensor configuration after igniting a gas mixture of 28.5% vol. hydrogen in air on one end of a closed pipe at different initial pressures. (**a**) Signal acquired at 0 bar overpressure with white points indicating automatically detected signal onset of the pressure wave (yellow points are discarded as they belong to signal features caused by acoustic waves in steel) and fitted slope (white solid line); (**b**) signal acquired at 2 bar initial overpressure with white points indicating automatically detected signal onset of the pressure wave and white solid and dashed lines indicating fitted and manually plotted slopes before and after deflagration-to-detonation transition (DDT), respectively. Yellow points and yellow solid line show the signal onset and the fitted slope of signal features due to acoustic wave propagation in the pipe material.

Figure 10 shows filtered fiber-optically acquired data along the pipe after igniting a gas mixture of 28.5% vol. hydrogen in air at to different values of initial overpressure.

For ambient pressure, i.e., 0 bar initial overpressure, similar signal features are observed as in the experiments using propane in air as seen when comparing Figure 9b and Figure 10a. Particularly, propagation of the pressure wave is linear and the same data evaluation procedure is used. The velocity is deduced as (500 ± 10) m/s in accordance with the value determined by the point pressure sensors of 469 m/s.

At 2 bar initial overpressure a more complex signal shape is observed (Figure 10b). The automatically detected signal onsets may be clearly attributed to two different signals. For fitting and determination of propagation velocity only those points are considered which may be assigned unambiguously to one of the two acoustic signatures.

First, a higher acoustic intensity is noticed leading to much more pronounced signal features of the structure-borne waves (marked in yellow) in the filtered data so that automatic signal evaluation is possible—albeit with a rather high uncertainty. The deduced velocity amounts to (4850 ± 1360) m/s in agreement with former results.

Moreover, propagation of the pressure wave along the observed length of pipe is non-linear and may be roughly divided into two linear regions with different slopes. In the first region as seen from the place of ignition (on the right in Figure 10b, 197–247 bins) a velocity of (590 ± 40 m/s) is calculated from signal onsets (white points) and linear fitting (white solid line). The second region (on the left, 179–196 bins) exhibits a considerably smaller slope corresponding to a much higher propagation velocity of the pressure wave. As the number of position bins in the region is rather low, linear fitting is not applicable here.

Since the local pressure transducers are positioned in the region of the pipe where faster propagation is observed fiber-optically they may be used as reference. The velocity deduced from the measured raise in pressure is 2080 m/s. A line with corresponding slope is overlaid in the plot of the fiber-optic data in the second region (white dashed line) and shows an overall agreement with the gradient of the onset points.

## 4. Discussion

### 4.1. Model of Acoustic Waves Propagation in Pipes

**Figure 11:**
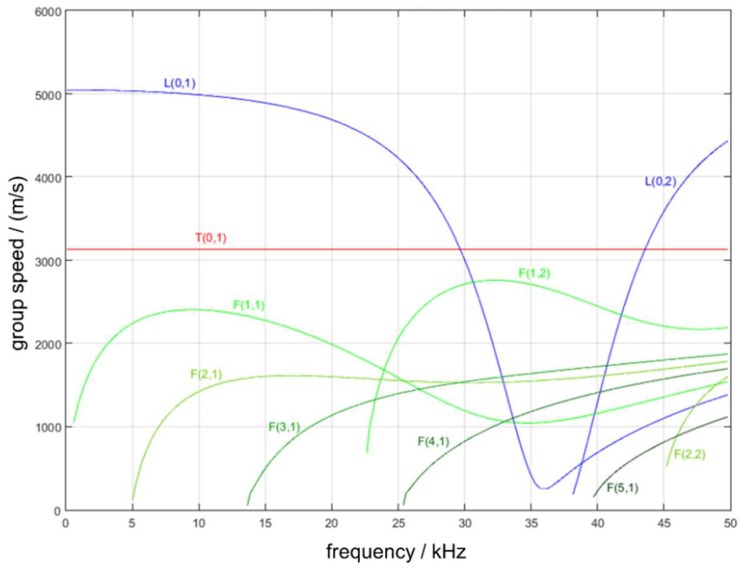
Dispersion curves of group speeds for different acoustic modes propagating in cylindrical waveguides determined by numerical simulation for the given pipe geometry and material properties.

For comparison of the propagation velocities of structure-borne acoustic waves inside the pipe material determined by distributed acoustic sensing to theoretical predictions, a numerical simulation of the propagation of acoustic waves was carried out for the chosen pipe geometry. The calculated dispersion curves for the different acoustic modes expected in a cylindrical waveguide are presented in Figure 11. As the frequency spectrum of the excitation acquired by fiber optic sensing is dominated by frequencies below 7 kHz (data not shown), group speeds are analyzed in the low-frequency region. The group speed of the longitudinal mode L (0,1), which is the fundamental mode in a cylindrical waveguide, amounts to 5047 m/s in the low frequency limit (bar speed). All the experimentally determined acoustic velocities of the main mode are in good agreement with this value.

Interestingly, the (frequency independent) group speed of the torsional mode T (0,1) is 3130 m/s indicating this as the second mode observed in some of the experiments with velocities in a range of 3000 m/s to 3300 m/s.

Recently, a study [34] was presented targeting the localization of CO_2_ leakage from transportation pipes. Propagation velocities of acoustic waves traveling in the pipe material were determined in a laboratory scale experiment on a steel pipe of comparable dimensions as the ones used here, i.e., 6 m length, 50 mm outer diameter, and 46 mm inner diameter, employing two acoustic emission sensors and Nielsen-Hsu pencil lead break tests as excitation source. Two main modes with velocities of 5070 m/s und 3268 m/s were derived in agreement with the findings of the present study.

### 4.2. Localization Accuracy and Precision

**Figure 12:**
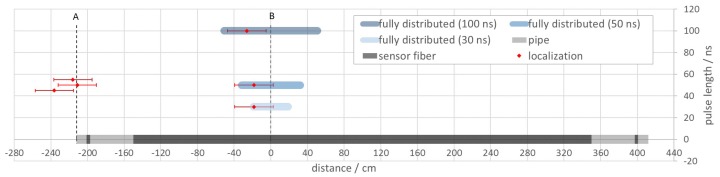
Comparison of localization accuracy and precision for a fully distributed and a quasi-distributed sensor configuration consisting of two localized sensor regions along a pipe. Points of disturbances (**A**,**B**) are indicated as dashed lines for different pulse lengths. Localization precision values of the fully distributed configuration (blue bars) are directly given by the spatial resolution corresponding to the pulse length. For the quasi-distributed setup fiber-optically localized positions of impact are indicated (red diamonds) with corresponding error bars representing the localization uncertainty. (For localization around A using 50 ns pulses, slight shifts were introduced to the value of the pulse length to improve clarity of the representation).

In the first set of experiments, which were carried out with a fully distributed sensor configuration, the determined position of the disturbance and the localization precision can be directly read from a spatial-temporal representation of raw or adequately filtered data. Localization accuracy is basically determined by the accuracy of the mapping of bins to actual positions along the pipe. This can be done in a calibration procedure after installation of the fiber where the fiber is tapped in crucial places and the observed signals are assigned to the position. This procedure may be carried out with the shortest pulse length available yielding an expected accuracy determined by the spatial resolution of the system. Here, this amounts to 1 m / 7.9 = 0.13 m along the pipe for 10 ns pulses and the chosen helical configuration of the fiber. For excitation on the side of the pipe, which allows for an estimation of deviations in both directions along the pipe, the experimentally determined localization precision is visualized relative to the actual position of impact in Figure 12 for the three different pulse lengths. It amounts to ± 51 cm for 100 ns, ± 32 cm for 50 ns, and ± 19 cm for 30 ns pulses.

When employing a quasi-distributed sensor configuration knowledge of the propagation velocity in the material is a prerequisite to map the observed events to a position on the pipe based on the time difference of signal onset. In practice, this value could be either taken from a theoretical consideration or from a calibration procedure with localized signals in known positions.

For the sake of analyzing localization accuracy here, the relative deviation of the experimentally determined speed values from the theoretical value confirmed by numerical simulation is used as a measure for the deviation from the actual point of impact. The localization accuracy is depicted in Figure 12 for both positions of impact (front face and side of the pipe). The corresponding localization precision was derived from the measurement uncertainty by error propagation. It amounts to ±20 cm to ±22 cm independent of the evaluation procedure or pulse length.

A summary of the deduced localization uncertainty for different sensor configurations and evaluation procedures is given in Table 2.

However, the positions of impact determined for the quasi distributed configuration by evaluation of signal onsets hints at an additional systematic deviation to one side of the points of impact corresponding to systematically underestimated time differences between the two localized sensors. This is also evident in the trend to somewhat higher speed values (Table 1) deduced by this method. Further refinement of the evaluation procedure, e.g., consideration of other signal features may mitigate this effect as can be seen in the much higher position accuracy achieved when employing correlation procedures for data evaluation.

Overall, the quasi distributed sensor configuration tends to produce smaller localization uncertainties, especially at longer pulse lengths than the fully distributed one for the setup with a short pipe segment used here. This is attributed to the possibility of signal averaging over a certain bin range and resulting higher signal-to-noise levels. However, for longer pipes it is expected that signal intensities and localization precision will deteriorate if the distance between disturbance and the localized sensors increases. This effect was also observed by Cui et al. [34] for localization on a pipe with acoustic emission sensors. For the fully distributed fiber-optic sensor configuration this effect is irrelevant so that the localization precision should be only influenced by the effect of overall signal attenuation along the optical fiber which is present for any sensor configuration.

For selection of an optimal application geometry in an industrial environment these advantages and disadvantages must be considered alongside issues concerning accessibility of pipes, overall range to be monitored and the required spatial resolution.

Moreover, for practical pipes, which often have a larger diameter than the ones inspected here, the localization precision and accuracy would increase when employing similar fiber pitch as in the experiments shown here. However, in this case the monitoring distance along the pipe would be decreased and/or the bandwidth restricted further. Therefore, it is expected to be favorable to adjust the fiber pitch to larger values in order to cover longer stretches of pipeline, as in many practical cases lower spatial resolution than the one reached here should be sufficient.

### 4.3. Monitoring of Gas Explosions in Pipes

In the set of experiments carried out to detect and localize propagating pressure waves in pipes it was found that the fully distributed fiber-optic sensor configuration is indeed able to track such waves continuously along the pipe. Besides the intended use in condition monitoring this opens new perspectives in the fundamental study of gas explosions.

In general, gas explosions can be subdivided into two groups with tremendous differences in their consequences on pipeline structure. While deflagrations are characterized by velocities below the speed of sound in the medium and explosion pressures on the order of 10 bar above initial pressure, detonations propagate with supersonic velocities and pressures an order of magnitude larger usually causing catastrophic damage. Due to the complex interplay between fluid dynamics, heat transfer and turbulences depending on the specific gas mixture and pipeline configuration, determination of occurrence and location of deflagration-to-detonation transitions (DDT) is still a topic of current research [37,38].

Here, both measurement techniques, DAS and piezoelectric pressure transducers, confirmed a (constant) velocity in the range of the acoustic speed inside the medium after ignition of a gas mixture of propane in air. Together with the observed pressure values of $p_{\mathrm{ex}}\;-\;p_{0}=\;13\;\mathrm{bar}$, with *p*_ex_ being the explosion and *p*_0_ the initial overpressure, this is characteristic of a deflagration. A similar situation was found in the experiment with a gas mixture of hydrogen in air ignited at ambient pressure.

The advantage of using a fully distributed sensor as represented by the distributed acoustic sensing system, emerges in cases where a non-linear propagation takes place. This is illustrated in the experiment where a gas mixture of hydrogen in air was ignited at 2 bar initial overpressure. The supersonic propagation velocity and pressure values of $p_{\mathrm{ex}}\;-\;p_{0}=\;67\;\mathrm{bar}$ and 83 bar, registered by the two piezoelectric pressure transducers, are indicative of a detonation. Due to their positioning it is however not possible to capture the point of DDT. A large number of closely spaced conventional point sensors would be necessary to generally determine this point with sufficient spatial resolution. Only the fully distributed system enables observation of this point without a priori knowledge of its location.

Therefore, DAS, together with an appropriate sensor configuration, may be used as tool for fundamental studies on the behavior of gas explosions in pipes. As shown in the first part of this paper, the present helical sensor configuration may allow to localize such events with a spatial resolution down to ~20 cm. This would correspond to installing 30 piezoelectric pressure transducers on a pipe of 6 m length to achieve the same resolution.

## 5. Conclusions

In the present study, the capability of DAS to detect and localize transient events on pipes was investigated. It was found, that in conjunction with a fitting sensor configuration of the optical fiber, mechanical impacts can be identified and localized along the pipe. Depending on the favored application, a fully distributed configuration, represented by continuous helical wrapping of the sensor fiber around the pipe or a quasi-distributed configuration consisting of short local sections of densely wound fiber, may be favorable. The localization accuracy and precision in general depends on the chosen fiber configuration, experimental settings (i.e., pulse length) and the procedure of data evaluation. For the parameters tested here, values down to 20 cm were achieved. In summary, the methods presented in this study may be understood as a toolbox for practical implementation of DAS on pipes.

In a second set of experiments the technique was applied for spatially continuous tracking of pressure waves in pipelines following gas explosions. It was demonstrated that the employment of DAS with a fully distributed sensor configuration not only allows for observation of propagation with a constant velocity but also of accelerated motion. In contrast to conventional testing of gas explosions with local pressure sensors, the proposed technique may provide a means to identify the point of deflagration-to-detonation transition without gap and without a priori knowledge of its location.

The approach is therefore of interest not only in condition monitoring of pipes but also in investigations of the behavior of gas explosions in closed pipe systems.

## Figures and Tables

**Table 1:** Summary of acoustic propagation velocities (main mode) obtained by distributed acoustic sensing (DAS) using different sensor configurations, optical pulse lengths and evaluation procedures. Positions of mechanical impacts are either on the front face (A) or on the side of the pipe (B). Uncertainty is given as twice the standard deviation (confidence interval of 95.5%).

No.	Sensor Design	Position of Impact	Pulse Length/ns	Evaluation Procedure	Velocity/(m/s)	Uncertainty/(m/s)
1	fully distributed	A	100	differentiation, signal onset (automatically)	5370	190
2	fully distributed	B	100	differentiation, signal onset (automatically)	4620	520
3	fully distributed	B	50	differentiation, signal onset (manually)	5100	-
4	fully distributed	B	30	differentiation, signal onset (manually)	5100	-
5	quasi distributed	A	50	differentiation and summation, signal onset (automatically)	5320	180
				differentiation and summation, correlation (absolute maximum) (automatically)	5040	170
				differentiation and summation, correlation (mean over relative maxima) (automatically)	5080	170
6	quasi distributed	B	100	differentiation and summation, signal onset (automatically)	5710	210
7	quasi distributed	B	50	differentiation and summation, signal onset (automatically)	5510	200
8	quasi distributed	B	30	differentiation and summation, signal onset (automatically)	5510	200

**Table 2:** Summary of the localization uncertainties obtained in DAS measurements for different sensor configurations, optical pulse lengths and evaluation procedures. Positions of mechanical impacts are either on the front face (A) or on the side of the pipe (B). Uncertainty is given as twice the standard deviation (confidence interval of 95.5%).

No.	Sensor Design	Position of Impact	Optical Pulse Length/ns	Evaluation Procedure	Localization Uncertainty/cm
1	fully distributed	A	100	differentiation, direct	51
2	fully distributed	B	100	differentiation, direct	51
3	fully distributed	B	50	differentiation, direct	32
4	fully distributed	B	30	differentiation, direct	19
5	quasi distributed	A	50	differentiation and summation, signal onset (automatically)	20
				differentiation and summation, correlation (absolute maximum) (automatically)	20
				differentiation and summation, correlation (mean over relative maxima) (automatically)	20
6	quasi distributed	B	100	differentiation and summation, signal onset (automatically)	22
7	quasi distributed	B	50	differentiation and summation, signal onset (automatically)	21
8	quasi distributed	B	30	differentiation and summation, signal onset (automatically)	21
